# Education Research: Targeting Self-Described Knowledge Gaps to Improve Functional Neurologic Disorder Education Among Clinicians

**DOI:** 10.1212/NE9.0000000000200239

**Published:** 2025-09-05

**Authors:** Richard Miller, Sarah Lidstone, David L. Perez, Dara V.F. Albert

**Affiliations:** 1 Department of Pediatrics, Division of Neurology, Nationwide Children's Hospital/The Ohio State University, Columbus;; 2 Integrated Movement Disorders Program, University Health Network, Toronto, Canada;; 3 Division of Neurology, Temerty Department of Medicine, University of Toronto, Canada; and; 4 Departments of Neurology and Psychiatry, Mass General Brigham Integrated Healthcare System, Massachusetts General Hospital, Boston.

## Abstract

**Background and Objectives:**

The objective of this study was to improve functional neurologic disorder (FND) education by identifying knowledge gaps among providers who registered for an online course on FND. The field of FND is rapidly evolving with new frameworks for understanding the diagnosis, pathophysiology, and treatment. This leads to the potential for knowledge gaps among clinicians who care for patients with FND. The shift away from terminologies such as “psychogenic” or “conversion” disorders underscores advances in how FND is conceptualized. Yet, gaps in the assimilation of this new knowledge among medical providers have been consistently found in surveys. This study is a qualitative analysis, allowing participants to state their specific knowledge gaps and identify content areas most in need of additional education.

**Methods:**

Providers from various disciplines including neurologists and other physicians, psychologists, and physical therapists enrolled in a virtual course containing 9 asynchronous lectures on various FND topics followed by 2 live webinars (fndsociety.org/fnd-education/virtual-education-course). Participants were invited to optionally submit questions for the live webinars to the expert panel about the care of FND in various treatment settings. A qualitative descriptive research design was used, with conventional content analysis applied to identify themes from participant questions.

**Results:**

One hundred ninety-one responses were collected from 268 participants over 2 months for a 71% response rate. Participant responses clustered on specific clinical presentations (e.g., functional seizures [FSs]), communication challenges with patients and other providers, inpatient challenges (e.g., when admission might be warranted), and outpatient challenges, such as limited access to multidisciplinary teams. Some participants explicitly stated outdated attitudes about FND.

**Discussion:**

Qualitative analysis of the participant responses revealed priority areas of knowledge gaps, indicating potential underexplored avenues for high-impact education on FND. These areas include diagnostic uncertainty, such as the presence of comorbid medical illness, FSs, and tools to help the patient when best practice models are not available. Developing case-based learning to better foster illness scripts and modules on psychoeducation and psychological treatments for the nontherapist FND provider would enhance existing educational tools to allow providers in every setting to better care for patients with FND.

## Introduction

Functional neurologic disorder (FND) is a condition at the intersection of neurology, psychiatry, and rehabilitation specialties, whereby patients have sensorimotor symptoms demonstrating features of internal inconsistency. This condition is common across outpatient,^1^ inpatient, and emergency department care settings^2^ and has been described in medical literature for well over a century.^3^ Yet, perspectives on FND have evolved over time. Rather than a singular mechanistic focus on affectively charged psychological experiences being “converted” into bodily symptoms, FND is now believed to represent a biopsychosocially complex brain disorder that is driven predominantly by aberrant communication across neural networks.^4-6^ In line with the updated understanding that FND is mechanistically and etiologically heterogeneous, a diverse set of biologically relevant psychopathologic constructs are believed to be important in the development and maintenance of FND, including biased attention processing, impaired action-authorship perceptions, alexithymia, dissociation, fear avoidance, and aberrant predictive processing among others.^7,8^ Adverse life experiences, although noteworthy for some patients with FND, are not universally found (and/or relevant) for all individuals in this diagnostic category.^9^ The shift in terminology away from “psychogenic” or “conversion” disorder toward FND underscores this change in thinking.^10^

Multiple studies across various regions and cultures have documented a lag in provider understanding of an updated conceptualization of FND.^11-14^ The proportions of providers surveyed who indicate that FND is the result of stressful events, is exclusively a psychiatric disorder, or is feigned, show that implicit bias against the legitimacy of FND or explicit bias against patients with FND (“time consuming,” “difficult”) are close to 40% in some studies.^15,16^ These numbers are alarming, given the importance of consistent messaging and education from all providers in reinforcing the diagnosis of FND.^17^ Some respondents also report unease around making a diagnosis of FND for fear of missing another medical cause of symptoms,^18^ although the rate of misdiagnosis is commonly held at 4%, and experts maintain that FND should not be treated as a diagnosis of exclusion.^1^

Effective patient education has been shown to reduce unnecessary health care utilization,^19^ and acceptance of an FND diagnosis is considered to be a facilitator of treatment success in surveys of neurologists^14^ and clinical outcomes studies.^20^ Bias against patients with FND correlates with reduced referrals to physical therapy (PT) and cognitive behavioral therapy (CBT)^21,22^ that have been shown to be beneficial. This reinforces how improving provider background knowledge on FND would improve patient care. Yet, providers report receiving insufficient education on FND during training in survey studies.^22,23^

Many authors have decried the lack of formal, structured education on FND in medical training.^22^ Reported educational interventions for providers on FND emphasized the role of multidisciplinary teams (MDTs), availability of effective therapies, and use of updated brain-based mechanistic models.^8,24^ Previous studies designed to describe the knowledge gap have been surveys.^11-14,25^ These quantified the frequency of negative or obsolete attitudes about FND often among neurology specialists. However, the quantitative design limits those studies to guessing at the knowledge gaps and biases that the study designers assume respondents might have. This study is a qualitative assessment of free-text responses, addressing a deficit in the existing literature by allowing a broad cohort of clinicians to detail what their knowledge gaps actually are. This information can drive the creation of higher impact FND curricula.

## Methods

A qualitative descriptive research design was used. Providers from various disciplines including neurologists, psychologists, psychiatrists, physiatrists, occupational therapists (OTs), PTs, and speech and language pathologists (Table 1) enrolled over 2 months in an online virtual course on FND offered by the FND Society (FNDS). The course consisted of 9 asynchronous lectures on various FND topics followed by 2 live webinars, “managing outpatient clinical challenges in FND care” and “emergency department, epilepsy monitoring unit, and acute hospitalization challenges in FND care”. On registration for the course, participants were invited to optionally submit questions for the live webinar panels (Figure).

**Table 1:** Respondents by Profession

Respondent role	Number of respondents	Proportion of total respondents
Neurologist	79	0.29
Psychiatrist	29	0.11
Physical medicine and rehabilitation (physiatrist)	11	0.04
Other physicians	8	0.03
Total physicians	127	0.47
Psychologist	46	0.17
Occupational therapist	8	0.03
Physical therapist	36	0.13
Speech and language pathologist	6	0.02
Nurse	2	0.01
Other/no response	43	0.16
Total	268	

**Figure:**
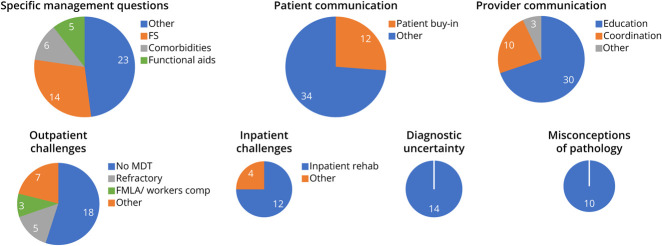
Coded Responses by Theme Depiction of the themes among the coded responses by frequency of appearance. Each circle represents 1 code and the subthemes within it. Sizes of the circles are adjusted to roughly represent the relative frequency of which each theme was encountered. FMLA = Family Medical Leave Act; FS = functional seizures; MDT = multidisciplinary team.

One team member (R.M., male, child neurology resident) reviewed and initially coded the open-ended participant questions for emergent themes using conventional content analysis. At each stage, authors R. Miller and D.V.F. Albert (female, child neurology attending) met to ensure that memo-keeping was documented and all themes were credible. Conventional content analysis generates codes from within the data rather than applying preexisting theory, highlighting the difference between this and previous survey studies.^23^ The responses from both live webinars were first evaluated independently, and codebooks were generated to assess the data for patterns. Then, a single codebook was compiled to consolidate the codes from each session. Last, the responses from both sessions were combined and re-coded using the consolidated codebook to ensure constant comparative analysis.

### Standard Protocol Approvals, Registrations, and Participant Consents

The Institutional Review Board at the Research Institute at Nationwide Children's Hospital evaluated the study (STUDY00003691) and deemed it not human research and exempt from needing consent from participants.

### Data Availability

Anonymized data not published within this article will be made available by request from any qualified investigator.

## Results

A total of 268 participants enrolled in the course who submitted 191 questions for panelists, resulting in a 71% response rate (Table 2).

**Table 2:** Codes and Example Responses

1. Specific management questions	In my experience in pediatric neurology, most patients with PNES (psychogenic nonepileptic seizures) have convulsive episodes. Do you find that staring spells are less often due to PNES and besides obtaining an EEG, are there other clues you might use to distinguish between epileptic and non-epileptic spells?
	What is the role for short-term benzodiazepine use in terminating long-lasting dissociative seizures? There is probably some way that these medications relax muscles and address anxiety in a way that can break the feedback loop
	For cognitive complaints associated with FND, what is the role of compensatory strategies in treatment? For example, it might be helpful to train a client to use external memory aids to compensate for memory lapses. However, we often see over-reliance on these aids and a lack of confidence in cognitive abilities
2. Communicating with patients	How does rhetoric change in delivering diagnosis of possible FND in the acute emergency department (ED) setting vs outpatient setting (given factors of time limitations, patient anxiety in emergency settings, etc.)
	How would you recommend responding if/when patient's ask what causes functional symptoms from a neurological standpoint. More specifically, what areas or regions of the brain are involved? Also, in terms of the 3 P's (predispositing, precipitating, perpetuating biopsychosocial factors), is there typically a psychosocial stressor that can be easily/obviously identified?
	What is the best way to explain the diagnosis to patients so it is validating and supportive?
3. Communicating with providers	Are there any recommendations on how to best to correct misunderstandings of FND to more established attendings who are teaching their residents incorrect FND diagnostics
	How can we inform ER (emergency room) staff: docs, nurses, ambulance attendants about the appearance of stroke-like symptoms, and non-epileptic seizures, that may not need Ativan (lorazepam), or a hot-stroke protocol, but rather an approach that weaves neurology/psychiatry together with trauma-informed, collaborative care
	How to effectively and efficiently provide education regarding functional neurologic symptom disorder (FNSD) to a large group of medical professionals to allow for standardized assessment and care?
4. Outpatient challenges	Do you have any tips for managing FND in a resource constrained setting without access to a full MDT (multidisciplinary team)?
	How to navigate FMLA (family medical leave act) and disability for FND patients
	Patients with FND often treated like a “hot potato.” No one sees patient as falling within their mandate. Thoughts on how to generate interest in/committment to helping these patients without becoming the identified provider for every one of them?
5. Inpatient/emergency department challenges	As a hospital-based outpatient physical therapist, I see people with FND, but who are not diagnosed as such by a physician. In one of my conversations with a neurologist, they maintained that they want their inpatient neurologists to be highly sensitive to emergent “organic” pathology, so they are not set up to diagnose FND in an emergent setting. Can you provide 2–3 examples where an inpatient neurologist was able to diagnose FND, and the difference in how the patient was managed and responded based on that ability to diagnose?
	What is current best practice for triage criteria? Who benefits from multidisciplinary rehab?
	Some patients seem to take on the FND label and develop health system dependence and pathological illness behaviour, leading to recurrent inpatient presentations? What to do? There must be not just acceptance of a label but key concepts of what FND is?
6. Diagnostic uncertainty	If someone has a diagnosis of FND or known to have a “functional overlay” to their presentation, how should we approach investigation of new symptoms? For example, ordering neuroimaging, blood work, or other tests if there are positive signs of FND.
	How many “second opinions” do you need to be sure about the functional diagnosis?
	Is there any caveat to diagnosing FND immediately and straight to the point in an emergency setting? In the sense of possibly underlying/luxating structural disorders that may emerge later on
7. Misconceptions of pathology	Many patients are anxious when learning about FNDS. Is there any indication for a psychotropic medication in ER, for patients with FNDS?
	What is the role for short-term benzodiazepine use in terminating long-lasting dissociative seizures? There is probably some way that these medications relax muscles and address anxiety in a way that can break the feedback loop of ongoing movements, but I worry about the precedent it sets for (1) promoting an idea that we are using these for their anticonvulsive effect and (2) encourages reliance on these as a medication of choice for longer periods. Do you have thoughts on this?
	What patient experiences trigger the need for emergency and inpatient care?

Abbreviation: FND = functional neurologic disorder.

Examples are direct quotes from respondents and may include any spelling or grammatical errors made by the respondent.

### Theme 1: Specific Management Questions

These responses made up 25% of the total and indicated specific FND practice questions. There were 3 major subthemes in this code: functional seizure (FS) management, comorbid diagnoses, and the use of assistive devices. Participants inquired about the optimal way to treat FSs in the acute and outpatient settings, how to distinguish functional from epileptic seizures, and the risks of treating or not treating a seizure with emergency medication. Moreover, responses regarding FSs were more likely to ask about how to manage an acute episode and the risks and benefits of administering treatment for an epileptic seizure than responses discussing other FND subtypes. Some of the motivation behind wanting to treat a seizure was related to a belief that the anxiolytic properties of benzodiazepines would also be of potential benefit for a FS. Regarding assistive devices, many included their supposition that prescribing them reinforces disability rather than recovery and asked about the balance of safety when fall risks remain or how to approach patients who request a wheelchair. In addition, participants asked about the role of investigations, such as neuroimaging, especially when “organic” disease is also present, such as functional weakness in acute inflammatory demyelinating polyneuropathy.

### Theme 2: Communicating With Patients

This code applied to 25% of responses. The most frequent concern was establishing diagnostic buy-in. Some medical settings may not be conducive to a validating conversation, and providers reflected a need to “improve the culture of emergency departments.” However, they noted challenges with patients who arrive with many previous investigations already performed and seeking additional opinions. On the outpatient side, responses recognized how the lack of patient diagnostic acceptance can create the care-seeking cycle that acute care providers noted. Other communication challenges identified were discussing FND diagnosis with family members and how to counsel patients when their symptoms evolve or flare up. A few responses were about changing technology, such as social media and artificial intelligence chatbots. Respondents noted that these tools changed patient expectations about their workup and treatment and wondered whether they were also affecting patient presentations in themselves.

### Theme 3: Communicating With Providers

This code applied to approximately 20% of responses. In this theme, providers sought to coordinate with each other, often from the perspective of an outpatient FND specialist trying to communicate their encounters with patients with other professionals. Specific professional roles mentioned include a spectrum of touchpoints for patients with FND, including school staff, primary care physicians, emergency department providers, and inpatient specialists. Emergency services were most frequently reported as a desired target of education, often specifically to prevent unnecessary evaluations and treatments for stroke or seizure. However, there were also many comments regarding inpatient neurologists, therapists, and specialists both seeking to correct outmoded assumptions and wondering how the primary provider for FND can best communicate with other specialists to ensure that a plan of care is followed for investigating new or recurrent symptoms.

### Theme 4: Outpatient Challenges

This code applied to approximately 20% of responses. There were 3 subthemes in this code: practicing without a multidisciplinary team, patients who fail to improve, and workers' compensation and Family Medical Leave Act (FMLA). An ideal model of FND care in the literature includes an MDT with a physician, physical and/or OT, and psychologist to help manage symptoms. Each discipline echoes the diagnosis of FND to reinforce diagnostic buy-in and recovery. Yet, many respondents experienced lack of access to multidisciplinary providers. The barriers to forming an MDT include a lack of interested allied professionals, as well as financial constraints from health care institutions in establishing the clinic and for patients in following up with multiple providers. Some providers specifically requested tailored education on CBT for FND that they could administer without referral, to work around the lack of available therapists. Some providers who had access to an MDT and FND-directed therapies such as COgnitive behavioural therapy for adults with Dissociative SEizures also struggled with patients who failed to improve. Providers also wondered how to go about approving financial resources for patients with FND, such as FMLA and workers' compensation.

### Theme 5: Inpatient Challenges

This code applied to approximately 8% of responses. Responses in this group specifically revolve around a source of contention in FND management: admission to a hospital inpatient ward, especially for the patient who presents repeatedly. Providers asked generally whether inpatient is the appropriate setting for care, wondering whether it was even within the mandate of inpatient neurologists to deliver an FND diagnosis, or merely to rule out “can't-miss” structural pathology. When admitting to the hospital, respondents asked what service they should go to: general medicine, a neurology primary service, or inpatient rehabilitation. Some providers specifically asked about how to avoid admitting at all, or if admitting, how to reduce length of stay, for patients who frequently engage with acute care services.

### Theme 6: Diagnostic Uncertainty

This code was used in approximately 7% of responses. The responses indicated a lack of provider comfort making a new FND diagnosis or relying on a previous one to explain symptoms. Providers sought concrete differentials to rule out when making an FND diagnosis. The codes in this section also deal with situations in which diagnostics are ongoing and providers seek clarification on when it is appropriate to communicate an FND diagnosis even when results are pending. A particular area of concern was patients with known FND who present with new symptoms.

### Theme 7: Misconceptions of Pathology

This code applied to approximately 7% of responses and denotes those asking about or implying a belief in a psychological event or theory component of FND pathology not supported by the current literature. Multiple providers asked about anxiolytic interventions for acute FND presentations. Other responses asked about the role of recent or childhood psychological trauma in FND presentations, in a way assuming such was key to an FND presentation.

## Discussion

The aforementioned themes interweave several noteworthy trends. First, knowledge gaps clustered in a few domains: comorbid medical illnesses and diagnostic uncertainty, indications for assistive devices, and management of FS. Second, many of the aforementioned themes evoke a lack of an illness script for FND. An illness script is a cognitive representation of the pathology, pertinent findings, treatment, and expected course of an illness.^26^ Querying a benzodiazepine as an anxiolytic for FND as in Theme 7 suggests a misunderstanding of pathology and evidence-based treatments. Questioning in Theme 2 on imaging, blood work, and other diagnostics reflects confusion or hesitation over the pertinent findings and a sense of treating FND as a diagnosis of exclusion. Finally, in Theme 3, on asking about the appropriate consultants and settings to deliver a diagnosis of FND, providers implied a lack of knowledge about treatment and expected course of FND. Medical education research has shown that illness scripts enable rapid comparison of a presenting patient with cognitive models to achieve a working diagnosis based on how closely the patient adheres to the script. The maturation of these scripts has been linked to the progression of trainees into clinicians and underlies the proliferation of case-based learning in medical education.^27^ Finally, regarding multidisciplinary outpatient care, it is clear from the responses in Theme 4 that many providers interested in FND encounter barriers to establishing a practice, from institutional financial barriers to a lack of interested or educated providers.

A scoping review^28^ was recently published of educational interventions for FND and related conditions, targeting education for health professional students and physicians. Their study identified 23 interventions—17 including a didactic lecture followed by a question-and-answer session, 14 including simulated patient encounters, and 12 including videos. The goals of the interventions varied. Nine interventions published from 1989 to 2007 (39% of articles) discussed “reattribution” training designed to help patients recognize links between emotions and symptoms, for example, the link between emotional stress, chronic muscle tightening, and tension headache. Four articles (17%) published between 2013 and 2023 emphasized communication skills for physicians without using a psychosomatic model of symptoms. Seven articles (30%) were specifically devoted to differentiating psychogenic non-epileptic events (PNEE) and epileptic seizures based on semiology or a specific open-ended interview format. Finally, 3 articles (13%) were didactics for management of FND, including delivering a diagnosis and making referrals. It is notable that interventions have shifted over time from the reattribution model to one that does not emphasize psychosomatic mechanisms, concordant with the evolution of FND pathophysiology literature, and future interventions should bring modern FND neuroscience to the forefront of didactics. Moreover, few published articles emphasize management strategies compared with articles focused on diagnosis and communication (Table 2).

To incorporate the findings of this study, previous interventions might be modified as follows. Education should reinforce an illness script by emphasizing that FND is not purely psychological, should not be treated as a diagnosis of exclusion, should introduce important positive signs such as Hoover sign or hip abductor sign, and should include follow-up of patients after initial diagnosis. Assistive devices should be addressed with a focus on not reinforcing disability, and consideration should be given to acute rehabilitation when patients are truly otherwise unsafe. Regarding FSs, while they have received special attention previously that our data suggest is warranted, topics should broaden beyond distinguishing functional from epileptic seizure semiology to include appropriate medical management and referrals. That management education may even go so far as to include tailored CBT teaching for the nontherapist FND provider, which would fill a keenly felt care gap in the availability of this treatment in many communities. Examples of educational interventions including the abovementioned modifications based off previously published models, are proposed further.

A recent intervention was reported that educated providers on how to deliver recent neuroscience advances on FND to patients, which could serve as a model for similar educational efforts.^24^ Their workshop included a preintervention survey and a presentation that reviewed a case of FND, diagnostic criteria, and economic costs of FND and also outlined common communication challenges. Next, they discussed recent neuroimaging findings that can be encapsulated for patients with FND to deliver the diagnosis in a supportive way and showed a simulated video demonstrating this approach. The session ended with further details on FND management, followed by time for questions and a postintervention survey. The authors themselves noted that future interventions would feature relevant physical examinations, consistent with this study. Further modifications of that intervention based on this study's findings would extend the patient's case to include outcomes, mention assistive devices, and include examples of CBT treatment for FND. There have also been reports of an Objective Structured Clinical Examination (OSCE) of a FS disorder encounter.^29^ The scenario consisted of a parent and a 16-year-old patient who was admitted to an inpatient neurology service and recently completed overnight continuous electroencephalography capturing an episode of FND. The parent and patient are written to be defensive, angry, and concerned about an underlying “organic” pathology and are specifically instructed to resist an FND diagnosis 3 times before acquiescing. The parent then asks how to respond to events at home and school, and the patient asks how to control them. Modifying this OSCE based on this study might take 2 forms: One might have the patient and parent insist on a more specific neuroscience-informed explanation of FND, along with a roadmap for referrals and expectations at each stage of treatment. Another option would be to switch to a case of functional lower extremity weakness and have the trainee rely on physical examination to make the diagnosis and then have the parent request assistive devices.

This study has several limitations. First, participants were sufficiently motivated and interested to register and participate in a virtual course on FND organized by the FNDS, likely representing a biased sample of clinicians. There may be additional or different knowledge gaps by clinicians who did not register for the course. Furthermore, questions posed by these respondents may not be fully reflective of unrecognized knowledge gaps. In addition, a different qualitative design recruiting participants for structured interviews until saturation was achieved could provide a different depth of perspective than this study, which samples from a broader audience on a more superficial level. This study could provide a basis for more in-depth characterization.

FND is a biopsychosocially complex condition that requires providers to demonstrate high confidence in their diagnostic and communication skills to convey a rule-in diagnosis to patients while also addressing any concurrent concerns regarding another serious medical/neurologic diagnosis. Performing unnecessary or repetitive workup risks reinforcing care-seeking behavior, and less knowledgeable providers may be less likely to refer patients to appropriate care. Updating the conceptualization of FND among all clinicians has accordingly become a priority of the field, and the FNDS, to better channel patients along a path toward symptom recovery. Based on our analysis of responses to a free-text prompt among providers who registered for an online FND education course, we found several fruitful domains to guide follow-up educational initiatives. These interventions would enhance the existing educational tools on MDTs to allow providers in every setting to better care for patients with FND.

## Glossary

CBT: cognitive behavioral therapy
FMLA: Family Medical Leave Act
FND: functional neurologic disorder
FNDS: FND Society
FS: functional seizure
OSCE: Objective Structured Clinical Examination
OT: occupational therapist
PT: physical therapy
